# HiFiAdapterFilt, a memory efficient read processing pipeline, prevents occurrence of adapter sequence in PacBio HiFi reads and their negative impacts on genome assembly

**DOI:** 10.1186/s12864-022-08375-1

**Published:** 2022-02-22

**Authors:** Sheina B. Sim, Renee L. Corpuz, Tyler J. Simmonds, Scott M. Geib

**Affiliations:** 1 USDA-ARS Daniel K. Inouye US Pacific Basin Agricultural Research Center, 64 Nowelo Street, Hilo, HI 96720 USA; 2 Oak Ridge Institute for Science and Education, Oak Ridge Associated Universities, Oak Ridge, TN 37830 USA

**Keywords:** PacBio HiFi, Circular consensus sequencing, Adapter, Sequence data filtering

## Abstract

**Background:**

Pacific Biosciences HiFi read technology is currently the industry standard for high accuracy long-read sequencing that has been widely adopted by large sequencing and assembly initiatives for generation of de novo assemblies in non-model organisms. Though adapter contamination filtering is routine in traditional short-read analysis pipelines, it has not been widely adopted for HiFi workflows.

**Results:**

Analysis of 55 publicly available HiFi datasets revealed that a read-sanitation step to remove sequence artifacts derived from PacBio library preparation from read pools is necessary as adapter sequences can be erroneously integrated into assemblies.

**Conclusions:**

Here we describe the nature of adapter contaminated reads, their consequences in assembly, and present HiFiAdapterFilt, a simple and memory efficient solution for removing adapter contaminated reads prior to assembly.

**Supplementary Information:**

The online version contains supplementary material available at 10.1186/s12864-022-08375-1.

## Background

The third generation of sequencing technology has ushered in a genome sequencing and assembly revolution in which genomes are being sequenced and assembled at an increasingly rapid rate. One current sequencing strategy is HiFi sequencing derived from high consensus accuracy circular consensus sequencing (CCS) on a PacBio Sequel II instrument. PacBio CCS sequencing leverages PacBio’s continuous long read technology to create a consensus sequence by sequencing a read repeatedly to produce a read pool with higher consensus accuracy than traditional long read technology and lengths that far exceed Illumina reads [1]. This technology has been used to sequence and assemble the breadth of eukaryotes, and is the preferred method used by various sequencing initiatives such as the Earth BioGenome Project [2, 3], the Vertebrate Genome Project [4], the i5K Initiative [5], the Ag100Pest Initiative [6], among others to generate data supporting highly contiguous and highly accurate contig assemblies that meet the criteria for reference-quality assemblies [7, 8].

Adapter filtering and read trimming are common aspects of pipelines analyzing Illumina short read data, with many existing tools [9, 10], and studies that characterize the impact of potential adapter contamination on assembly [11, 12]. Conversely, adapter filtering prior to assembly is not a common component of HiFi data analysis pipelines, with most HiFi compatible de novo genome assembly software tools suggesting the use of the output of PacBio CCS analysis as the input to the assembly software. However, in a survey of 55 publicly available SRAs of PacBio HiFi sequences, “PacBio Blunt Adapter” (UniVec database build 10.0, accession NGB00972.1) was found consistently in 53 out of 55 CCS datasets. A subset of these data were assembled using the three most common HiFi assembly programs (HiCanu [13], HiFiASM [14], and PB-IPA [15]), and integration of adapter sequence in the genomic contigs was detected in some of the final assemblies generated with each of the three assembly programs. To address adapter contamination, we present the software HiFiAdapterFilt [16], a simple and memory efficient adapter filtering approach developed to pre-process HiFi reads prior to assembly. We demonstrate this filtering and assembly using three of the 55 public SRA datasets (three insect species *Anopheles gambiae*, *Drosophila ananassae*, and *Vespa mandarinia*). Following processing through this pipeline, the resulting assemblies were free of adapter contamination, with no impact on the contiguity of the assembly, and in some cases correction of mis-joins caused by presence of the adapter in the input read dataset (Table 1). Based on these results, an adapter sanitation step for HiFi reads prior to assembly is highly recommended, and the consequences of the presence of adapter contamination in the pre-assembly read pool are discussed.

**Table 1:**
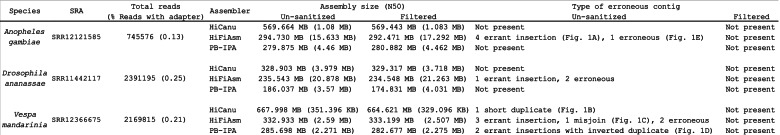
Public SRAs and types of errors produced by each of the three assembly programs

## Methods and implementation

Of the publicly available data on NCBI Sequence Read Archive (SRA), all available PacBio Sequel datasets of WGS HiFi reads, as of April 2^nd^ 2021, were selected for adapter contamination interrogation. All 55 raw fastq files were downloaded using SRA Toolkit v2.10.9 [17] and filtered using HiFiAdapterFilt v2.0.0 [16].

HiFiAdapterFilt was written in Shell to minimize dependencies and implements NCBI’s BLAST + v2.9.0 [18] to identify CCS reads containing adapter sequences that are then removed from the read pool prior to assembly. HiFiAdapterFilt accepts uncompressed fastq files, gzip compressed fastq files, and bam files (requires the program BamTools v2.5.1 [19]), which are all standard outputs of the PacBio SMRT Link software. If given no options, HiFiAdapterFilt will search all files of the appropriate file type in the working directory. Options include designations for the prefix, minimum length match, minimum percentage match, number of threads, and output directory (Table 2).

**Table 2:**
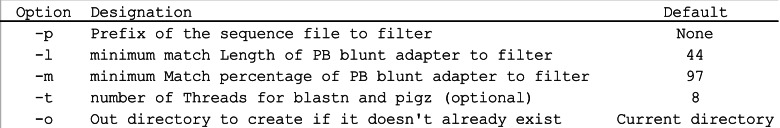
Options for HiFiAdapterFilt

HiFiAdapterFilt implements BLAST + to identify adapter-contaminated reads using the command `blastn` and the following options: `-task blastn -reward 1 -penalty -5 -gapopen 3 -gapextend 3 -dust no -soft_masking true -evalue 700 -searchsp 1,750,000,000,000 -outfmt 6.` BLAST + parameters were selected to mirror VecScreen [20] BLAST + parameters with a notable difference in the `dust` option. The resulting output is then filtered to return only reads containing matches 97% or greater and at least 44 bp out of the 45 bp Pacific Biosciences blunt adapter in length or 34 of the 35 bp Pacific Biosciences C2 primer. The `-l` and `-m` options for HiFiAdapterFilt allow for removal of shorter or less exact matches to the Pacific Biosciences Blunt Adapter.

For a subset of three insect taxa, the adapter contaminated raw HiFi reads were removed using `grep`, a command implemented by the HiFiAdapterFilt pipeline to create a filtered read set for assembly. The filtered read sets were then used as the input file for genome assembly using HiCanu v2.1.1 [13], HiFiASM v0.14 [14], and PB-IPA v1.3.2 [15]. 

A filtered read set was also created using the Cutadapt v3.4 [10] command line parameters `-b AAAAAAAAAAAAAAAAAATTAACGGAGGAGGAGGA;min_overlap = 35 -b ATCTCTCTCTTTTCCTCCTCCTCCGTTGTTGTTGTTGAGAGAGAT;min_overlap = 45 --discard-trimmed --revcomp -e 0.1` and assemblies were likewise generated using HiCanu v2.1.1, HiFiASM v0.14 and v0.15 where noted, and PB-IPA v1.3.2.

Assemblies were generated for all three taxa and all three data sets for each taxon (un-sanitized reads, HiFiAdapterFilt filtered reads, and Cutadapt filtered reads) using default parameters (commands in supplemental file). Assembly metrics were produced for each assembly using the BBmap [21] function `stats.sh` (S2) and the same BLAST + command was applied to the contig assemblies to identify adapter-contaminated contigs in the un-sanitized reads assemblies or validate their absence in the HiFiAdapterFilt and Cutadapt filtered assemblies.

HiFiAdapterFilt and Cutadapt were benchmarked using a compute node containing 2.40 GHz Xeon Platinum 8260 2nd Generation Scalable Processors containing 24 each, a total of 48 cores, and 384 GB of RAM. Though not identical, neither the HiFiAdapterFilt filtered genome and the Cutadapt filtered genome assemblies contained adapter contamination and were of similar final assembly size, N50, and total number of contigs for each species and assembly method (Table S2). Thus, all subsequent analyses comparing the un-sanitized assemblies with the filtered assemblies were conducted with the HiFiAdapterFilt filtered assemblies. Whole genome and local alignments between un-sanitized and filtered assemblies were performed using MUMmer4 [22] and BLAST + [18].

## Results and discussion

Screening 55 of the publicly available SRAs containing exclusively PacBio HiFi reads revealed adapter contamination in 53 of 55 datasets that were searched (Tab. S1). Analysis of CCS reads containing adapter sequence relative to the entire dataset revealed that adapter contamination was found disproportionately in extremely short reads as well as reads approximately 10 kb in length (Fig. S1A), and adapter contaminated reads had a slightly elevated GC content compared to uncontaminated reads (Fig. S1B). CCS reads containing adapter sequence predominantly fell into four types (Fig. S1C, Tab. S1), where adapters were located either at the 5’ end, internal to the read, at the 3’ end, or distributed throughout. An evaluation of the density and abundance of these read types across all datasets showed that CCS reads containing adapter sequence at the 5’ end were the most abundant, followed by reads with adapter sequence at the 3’ end, internal to the sequence, and the fewest reads contained adapter distributed throughout (Fig. S1D). A small subset of reads did not fall into any of these four categories and were not visualized. Of the 152,000 adapter sequences identified across all datasets, 89.6% were the reverse complement of the PacBio adapter sequence in contrast to the 10.4% that were the forward orientation. There is no clear pattern to the presence of these reads in the HiFi read datasets, and it is unclear why they are not trimmed or removed during subread generation and CCS analysis on the PacBio platform. Across all datasets screened, the proportion of reads containing adapter sequence never surpassed 0.25% and thus represents a low proportion of the overall data, so we recommend a strict removal of an adapter containing read, versus an attempt at trimming out the adapter region and trying to retain a portion of the read as trimming could result in retention of chimeric molecules or other contaminating factors.

Application of the HiFiAdapterFilt method implements BLAST alignment adapted from the VecScreen [20] pipeline to identify and remove adapter contaminated HiFi reads and generate a filtered read dataset free of PacBio adapter sequences. Other methods exist for adapter filtering with Cutadapt [10] being a popular tool. Cutadapt uses semi-global alignment methods to identify adapter sequences and was compared to the HiFiAdapterFilt method, yielding a similar but not identical filtered dataset. Evaluating assemblies generated with un-sanitized reads, HiFiAdapterFilt processed reads, and Cutadapt processed reads demonstrated adapter contamination in contigs assembled from the un-sanitized read sets but not in contigs assembled from reads that were filtered using HiFiAdapterFilt or Cutadapt (Tab. S2 and Tab. S3). Analysis of the adapter contaminated reads identified only by HiFiAdapterFilt or Cutadapt revealed that HiFiAdapterFilt detected proportionally more long reads (5 kb to 10 kb and > 15 kb) containing adapter sequences (Fig. S2) that are likely to have greater downstream effects on assembly. A comparison in computation speeds, wall time, and memory usage revealed that HiFiAdapterFilt was significantly more memory efficient than Cutadapt (Tab. S4). Both HiFiAdapterFilt and Cutadapt ran relatively quickly (though HiFiAdapterFilt used a greater amount of CPU and wall time) making both amenable to include in a sequence assembly workflow.

**Fig. 1:**
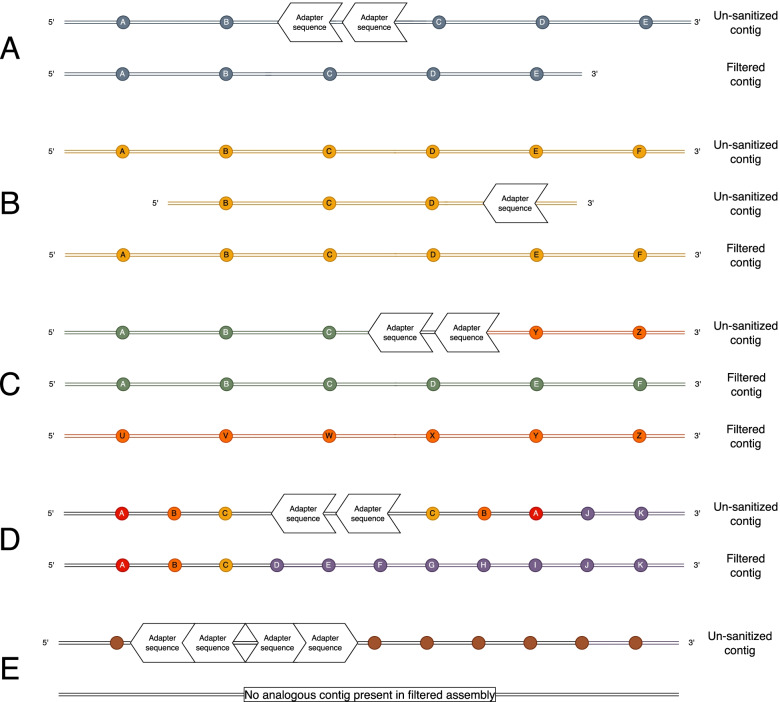
Schematic of the types of errors found in assemblies made from un-sanitized raw reads relative to their corresponding assemblies from filtered raw reads where all raw reads containing adapter sequences were removed. Five types of assembly errors were identified in the assemblies for the three taxa using three assembly programs: (**A**) errant insertions of adapter sequence in an otherwise contiguous contig with a near exact homolog in the corresponding filtered assembly, (**B**) short (truncated) duplicate contigs containing adapter sequence that is collapsed into a single contig in the corresponding filtered assembly, (**C**) mis-joined chimeric sequences which represent different parts of two non-homologous contigs in the corresponding filtered assembly, (**D**) contigs containing an inverted duplicate adjacent to the adapter sequence, and (**E**) contigs containing tandem adapter sequences where the adjacent sequence is not present in the filtered assembly

Screening of the un-sanitized assemblies of three insect species using three different publicly available HiFi assembly programs revealed assembled contigs containing adapter contamination. These erroneous contigs fell largely into in five types of error categories (Fig. 1). The first type was the errant insertion of adapter sequence where adapter-contaminated contigs in the assembly created from the un-sanitized read pool have a nearly completely homologous contig counterpart in the assembly created from the filtered read pool (Fig. 1A) where the adapter sequence is absent. The second type of error was the presence of a short or truncated duplicate contig containing adapter sequence on the end (Fig. 1B). The third type of error is a mis-join which resulted in a chimeric contig made up of parts from different contigs in the filtered assembly (Fig. 1C). The fourth type of error resulted in a duplicated inversion adjacent to the adapter sequence in a contig which otherwise had a completely homologous counterpart in the filtered assembly (Fig. 1D). The final, and most common type, of error were completely erroneous contigs that did not have a homologous counterpart in the filtered assembly (Fig. 1E). Each of the different assembly programs resulted in at least one of these errors, including HiCanu, which has a read trimming step as a component of its internal pipeline intended to remove regions potentially containing adapters (Table 1).

The NCBI software VecScreen is part of the NCBI genome assembly submission pipeline and screens all genomes submitted to NCBI for contamination by vectors in the UniVec database which includes the PacBio Blunt Adapter and C2 Primer (UniVec Build 10.0, accession NGB00973.1). If contaminant sequences are identified, their removal from final assemblies must be performed before they can be accepted for submission and released by NCBI. This can be achieved either by removing the entire adapter-contaminated contig, by excising adapter sequences and breaking the contig, or by masking those regions. These methods each have their own disadvantages. Removing whole contigs will reduce the completeness of the assembly when the errors are errant adapter sequence insertions or adapters with inverted duplicate sequences in otherwise accurate contigs (Fig. 1A and D) or in large chimeric sequences joined by adapter sequence (Fig. 1C). Conversely, excision of adapter sequence resulting in two smaller contigs will decrease the contiguity of assemblies containing contigs with errant adapter insertions (Fig. 1A) and will not remove truncated duplicate or completely erroneous contigs (Fig. 1B and E), though duplicate removal programs such as purge_dups [23], Purge Haplotigs [24], or HaploMerger2 [25] may be implemented to address the duplicate contigs separately. Simply masking the region can result in the retention of a mis-assembly and potentially chimeric sequence in the genome (Fig. 1C, D and E). As no method of adapter sequence removal after assembly is optimal and can result in a less complete, fragmented, or erroneous assembly, including a read-sanitation step prior to assembly will result in a more accurate and maximally contiguous genome assembly. When comparing which adapter screening method to use, one advantage of using an adapter screening method that utilizes BLAST searching is that it will find similar matches to the VecScreen pipeline that will be utilized during the assembly submission process by NCBI. HiFiAdapterFilt relies on the same local alignment methods employed by BLAST, so any subtle biases imposed by this alignment method versus other methods will be employed both during raw read adapter filtering steps as well as during the genome submission step in the genome assembly workflow. Additionally, the location of the adapter in the read is not impacted by this approach. Despite which filtering tool is used, a post-assembly screen for adapters prior to assembly finishing utilizing the UniVec screening parameters is highly encouraged to ensure that the assembly is sound and free of contaminants before investing downstream resources in completing the genome. Stringency of HiFiAdapterFilt can be easily adjusted most effectively by modifying the overlap length and percent match which are variables of the pipeline, with the caveat that over-relaxation of these parameters will remove legitimate reads with similarity to the PacBio adapters.

## Conclusions

Though a rare occurrence in all the datasets we evaluated, adapter-contaminated PacBio HiFi reads can result in assembly errors which include truncated duplicates, erroneous contigs, errant insertions of adapter sequence, mis-joins, and mis-assembly in the form of sequence inversions at the adapter insertion site. These assembly errors can easily be eliminated by performing a read sanitation step prior to assembly using publicly available tools such as Cutadapt [10], or HiFiAdapterFilt [16] which eliminated all instances of adapter contamination in the final assembly. Ideally, more stringent read filtering steps could be employed during subread generation and computation of circular consensus HiFi reads on the Sequel II system, either through application of one of the methods described here, or through modifications to the current adapter detection methods on instrument. Alternatively, read trimming and correction steps as a component of a genome assembly program can dramatically reduce the occurrence of adapter contamination, as demonstrated in HiCanu, which showed the lowest proportion of adapter contaminated contigs. Regardless, based on the data presented here, adapter filtering is highly recommended both on HiFi data derived directly from the sequencer as well as downloaded from the NCBI SRA to ensure clean datasets for downstream analysis.

## Availability and requirements

**Project name:** HiFiAdapterFilt

**Project home page:** https://github.com/sheinasim/HiFiAdapterFilt

**Operating systems:** Linux, MacOS, and Windows (using WSL)

**Programming language:** Shell

**Other requirements:** BLAST + v2.9.0, BamTools v2.5.1 (if starting with .bam file), and pigz (optional, for parallel gzip)

**License:** GNU General Public License v3.0

**Any restrictions to use by non-academics:** None

## Supplementary Information

**Additional file 1: Figure S1.** Summary plots of raw reads with Blunt Adapter sequences from 53 publicly available PacBio HiFi datasets.

**Additional file 2: Figure S2.** Density plot of read lengths and their proportions for adapter contaminated reads detected only by Cutadapt or HiFiAdapterFilt.

**Additional file 3: Table S1.** PacBio adapter location and abundance discovered in publicly available SRAs of HiFi data.

**Additional file 4: Table S2.** Contig statistics for three taxa using three different HiFi assembly software.

**Additional file 5: Table S3.** Number of adapter in unique contigs for all assemblies reported in Tab. S2.

**Additional file 6: Table S4.** Run statistics for HiFiAdapterFilt and Cutadapt on 3 SRA datasets.

## Data Availability

All raw data used is publicly available in NCBI SRA with accessions listed in manuscript. Commands and scripts to reproduce the results are included as a supplemental file.

## Abbreviations

NCBI: National Center for Biotechnology Information
USDA: United States Department of Agriculture
ARS: Agricultural Research Service
BLAST: Basic Local Alignment Search Tool
HiFi: High Fidelity
SRA: Sequence Read Archive
PacBio: Pacific Biosciences
i5K: Insect 5000 Genomes Initiative
