# Supplementary figures and images for: HiFiAdapterFilt, a memory efficient read processing pipeline, prevents occurrence of adapter sequence in PacBio HiFi reads and their negative impacts on genome assembly
Source: BMC Genomics. 2022 Feb 22;23:157. doi: 10.1186/s12864-022-08375-1 (PMC8864876; doi:10.1186/s12864-022-08375-1)

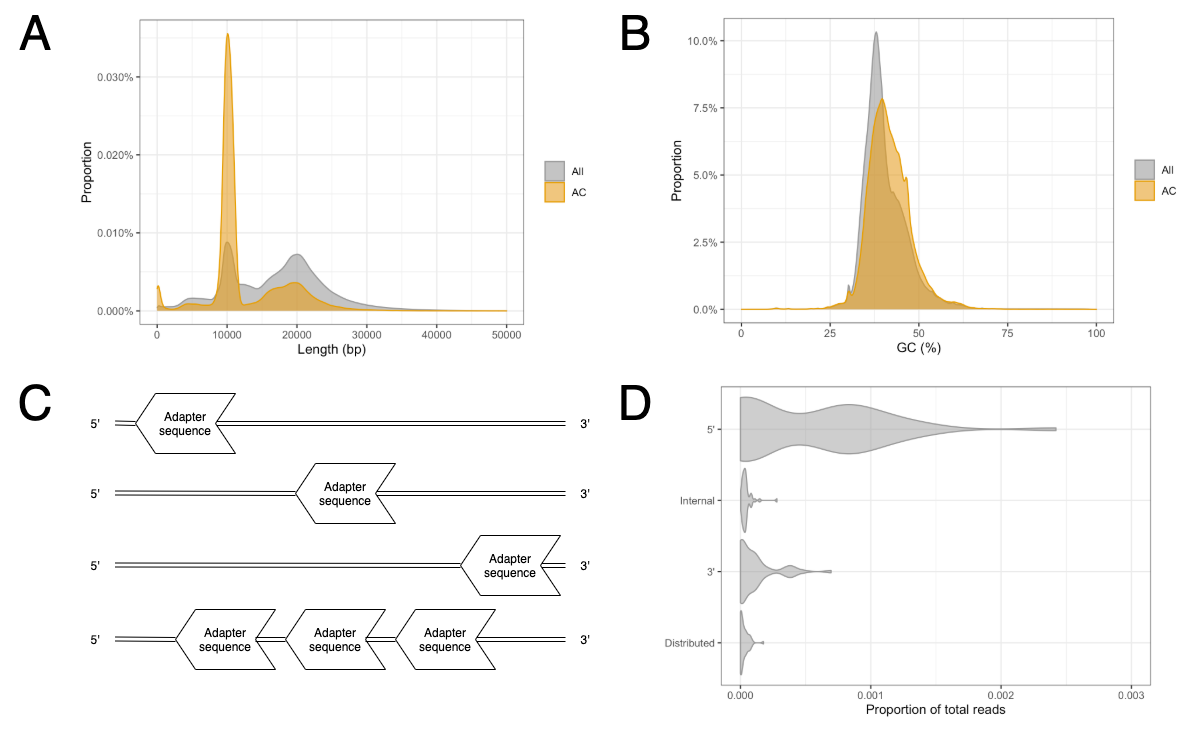

Supplement: Supplementary file 1 — Additional file 1: Figure S1. Summary plots of raw reads with Blunt Adapter sequences from 53 publicly available PacBio HiFi datasets. [file 12864_2022_8375_MOESM1_ESM.png]

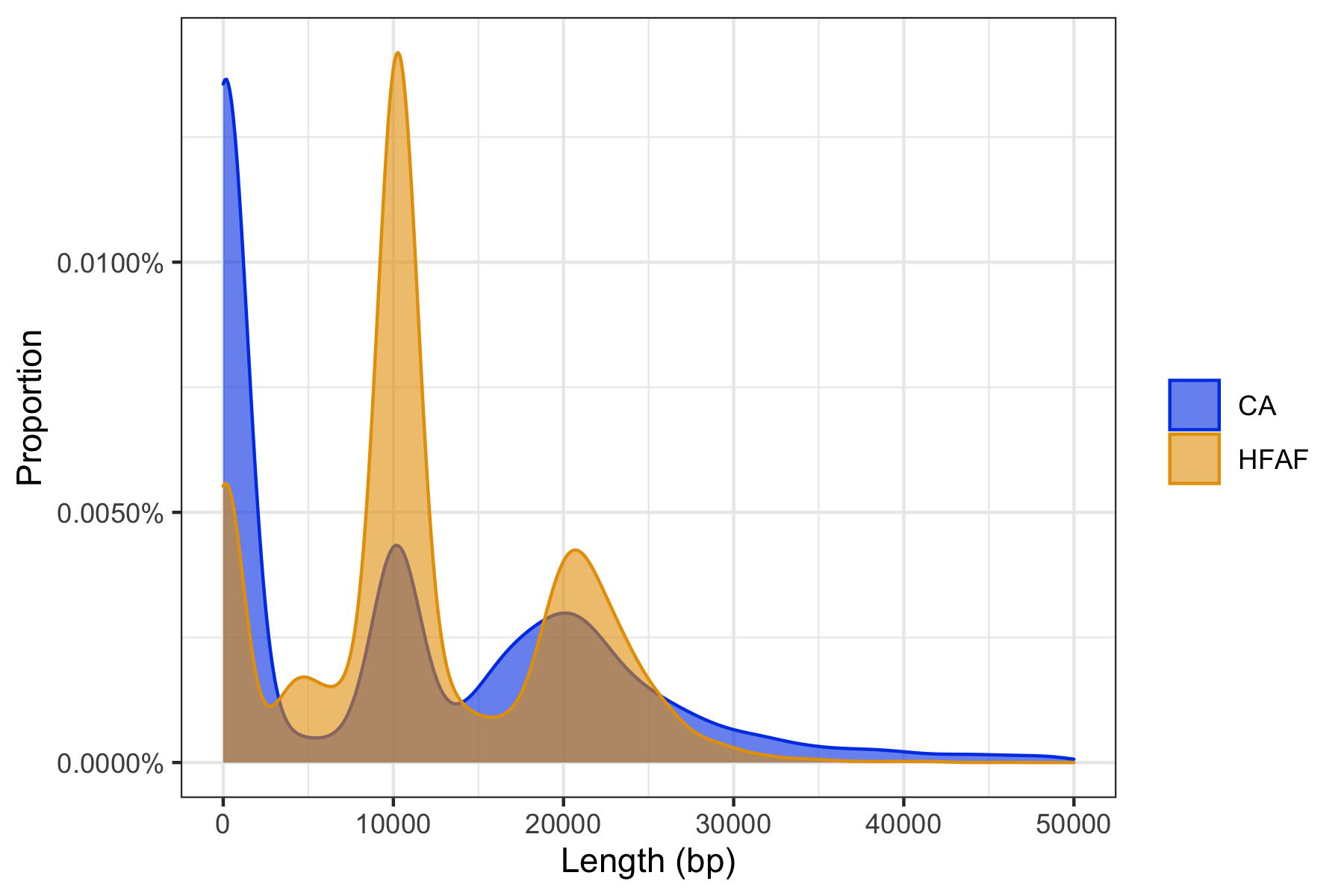

Supplement: Supplementary file 2 — Additional file 2: Figure S2. Density plot of read lengths and their proportions for adapter contaminated reads detected only by Cutadapt or HiFiAdapterFilt. [file 12864_2022_8375_MOESM2_ESM.png]
